# Low intensity pulsed ultrasound versus low-level laser therapy on peri-implant marginal bone preservation and soft tissue healing following dental implant surgery: a randomized controlled trial

**DOI:** 10.1186/s13005-025-00502-z

**Published:** 2025-04-23

**Authors:** Esraa S. Mahmoud, Amal M. Abd El-Baky, Osama M. Gouda, Hussein G. Hussein

**Affiliations:** 1 Department of Physical Therapy for surgery & burn, Faculty of Physical Therapy, Al-Hayah University in Cairo, Universities & Schools hub, New Cairo 3, 5th Settlement, Cairo, Egypt; 2 Department of Physical Therapy for surgery, Faculty of Physical Therapy, Cairo University, Cairo, Egypt; 3 Faculty of Dentistry, Badr University, Cairo, Egypt

**Keywords:** Dental implants, Low-intensity pulsed ultrasound, Low-level laser therapy, Osseointegration, Soft tissue healing, Pain, Oral health-related quality of life

## Abstract

**Background:**

Low-intensity pulsed ultrasound (LIPUS) and low-level laser therapy (LLLT) are proposed adjunctive therapies to enhance healing after dental implant surgery. However, direct comparisons of their effects on peri-implant marginal bone preservation and soft tissue healing remain limited. This randomized controlled trial aimed to compare the effectiveness of LIPUS and LLLT on peri-implant marginal bone preservation, soft tissue healing, pain levels, and oral health-related quality of life following dental implant placement.

**Methods:**

This single-blind, randomized controlled trial included 63 patients undergoing maxillary or mandibular implant placement, randomly allocated to LIPUS (*n* = 21), LLLT (*n* = 21), or control (*n* = 21) groups. LIPUS was applied twice weekly for 4 weeks, while LLLT was administered in 4 sessions over 2 weeks post-implant. Marginal bone loss (MBL) and OHRQoL (OHIP-14) were assessed at baseline, 6, and 12 weeks. Soft tissue healing (Landry Healing Index) and pain (VAS) were evaluated at baseline, 7-, 14-, 21-, and 30-days post-implant.

**Results:**

LIPUS significantly reduced marginal bone loss at 6 weeks and 3 months post-implant compared to LLLT and control groups (*p* < 0.05). LLLT demonstrated superior soft tissue healing at 7-, 14-, 21-, and 30-days post-implant (*p* < 0.05). Both interventions significantly decreased pain intensity and improved OHRQoL at various time points compared to the control group (*p* < 0.05).

**Conclusions:**

LIPUS and LLLT significantly enhance peri-implant marginal bone preservation, soft tissue healing, pain management, and OHRQoL in dental implant patients compared to standard care. LIPUS was more effective for peri-implant marginal bone preservation, while LLLT excelled in soft tissue healing.

**Trial registration:**

This study was registered at ClinicalTrials.gov (NCT05938868) on July 11, 2023.

**Supplementary Information:**

The online version contains supplementary material available at 10.1186/s13005-025-00502-z.

## Background

Tooth loss is a global health burden affecting millions worldwide [1]. An estimated 158 million people suffer from severe tooth loss globally [2, 3]. Increased life expectancy and oral health awareness [4] have elevated dental implant demand [5]. Dental implants provide reliable, long-term restoration of function, esthetics, and quality of life for edentulous patients [6].

Approximately 3 million dental implants are placed annually in the United States, with global figures reaching 12–15 million implants yearly [7]. Despite significant technological advancements, contemporary implants show success rates of 95.2% at 5 years and 89.4% at 10 years [8], meaning 5-10.6% still fail. Chrcanovic et al. [9] found that early failures within the first year primarily result from poor osseointegration or inadequate soft tissue healing (62.1% of failures), while late failures typically stem from peri-implantitis (28.6%) or biomechanical complications (9.3%). These failures significantly impact patient quality of life and generate substantial healthcare costs ranging from $3,000 to $12,500 per failed implant, depending on the complexity of subsequent reconstructive procedures required [10].

Osseointegration creates new bone around implants, enabling stable anchorage and masticatory force transfer [11]. Peri-implant soft tissue healing forms a mucosal seal, preventing bacterial infiltration and maintaining implant health [12].

Despite advancements in implant surface technology and surgical techniques, achieving optimal osseointegration and soft tissue healing remains a challenge [13, 14]. Compromised bone quality, systemic conditions, smoking, poor oral hygiene, and extended osseointegration periods impair healing, risking implant failure [15] and limiting early loading options [16].

Low-intensity pulsed ultrasound (LIPUS) and low-level laser therapy (LLLT have emerged as potential interventions to enhance implant-related healing [17–19]. LIPUS delivers non-thermal mechanical energy (30–100 mW/cm²) that triggers mechanotransduction pathways, upregulating crucial bone regeneration factors including BMPs, TGF-β, and IGF-1, while enhancing angiogenesis through VEGF stimulation [20–23]. Clinically, LIPUS has accelerated long bone fracture healing by up to 38% and improved non-union fracture rates by 67–90% [24, 25]. LLLT (photobiomodulation) uses red to near-infrared light (630–1000 nm) at low power densities (1-1000 mW/cm²) to interact with cellular chromophores, particularly cytochrome c oxidase, enhancing ATP production while modulating inflammatory responses by reducing pro-inflammatory cytokines and increasing anti-inflammatory mediators [26, 27].

Several studies have investigated the effects of LIPUS and LLLT on osseointegration and soft tissue healing. Iwai et al. [28] demonstrated that LIPUS enhanced osseointegration and reduced healing time in patients undergoing dental implant therapy. While, Liu et al. [17] found that LIPUS accelerated osseointegration and increased the maximal axial pull-out strength of titanium dental implants in a rabbit model. Similarly, a systematic review by Omasa et al. [29] concluded that LLLT could positively influence the early stages of osseointegration and improve implant stability, However, findings from randomized controlled trials have been variable. Regarding soft tissue healing, Palled et al. [30] reported that LLLT significantly improved healing parameters in implant patients, with their LLLT group showing lower modified sulcus bleeding index scores and probing depths at 6 weeks and 3 months post-operatively (*p* < 0.01).

Knowledge gaps persist as most studies examine LIPUS and LLLT independently, lacking direct comparisons in dental implant therapy. Comparative effectiveness research is essential to determine superior intervention outcomes. This controlled randomized study assessed the comparative outcomes between LIPUS and LLLT, evaluating their impact on dental implant osseointegration, surrounding soft tissue healing, post-operative pain, and patient-reported oral health-related quality of life.

## Methods

### Study design

This study was a randomized, controlled, single-blinded, parallel-group trial comparing the effects of low-intensity pulsed ultrasound (LIPUS) and low-level laser therapy (LLLT) on peri-implant marginal bone preservation and soft tissue healing in patients undergoing dental implant surgery. The study was conducted between July 2023 and September 2024.

### Ethical considerations

This trial is reported in accordance with the CONsolidated Standards Of Reporting Trials (CONSORT) statement [31] (Supplementary file 1), This study was conducted in accordance with the principles of the Declaration of Helsinki and was approved by the Research Ethical Committee of the Faculty of Physical Therapy, Cairo University (approval number P.T.REC/012/004647). The study was also registered on ClinicalTrials.gov (NCT05938868) before participant enrollment.

Participants provided written informed consent after receiving comprehensive study information. Participation was voluntary with unrestricted withdrawal rights. Data confidentiality was ensured through secure storage, restricted access, and anonymized publication of results.

### Settings

The study was conducted at a specialized dental clinic in Sheikh Zayed City, Giza, Egypt. This tertiary referral center features advanced equipment including digital radiography, intraoral scanners, and CAD/CAM technology. The facility comprises six dental operatories and dedicated sterilization and consultation areas. Staff includes specialized dental surgeons, periodontists, prosthodontists, and trained assistants, adhering to international infection control and safety standards.

### Participants

**Fig. 1:**
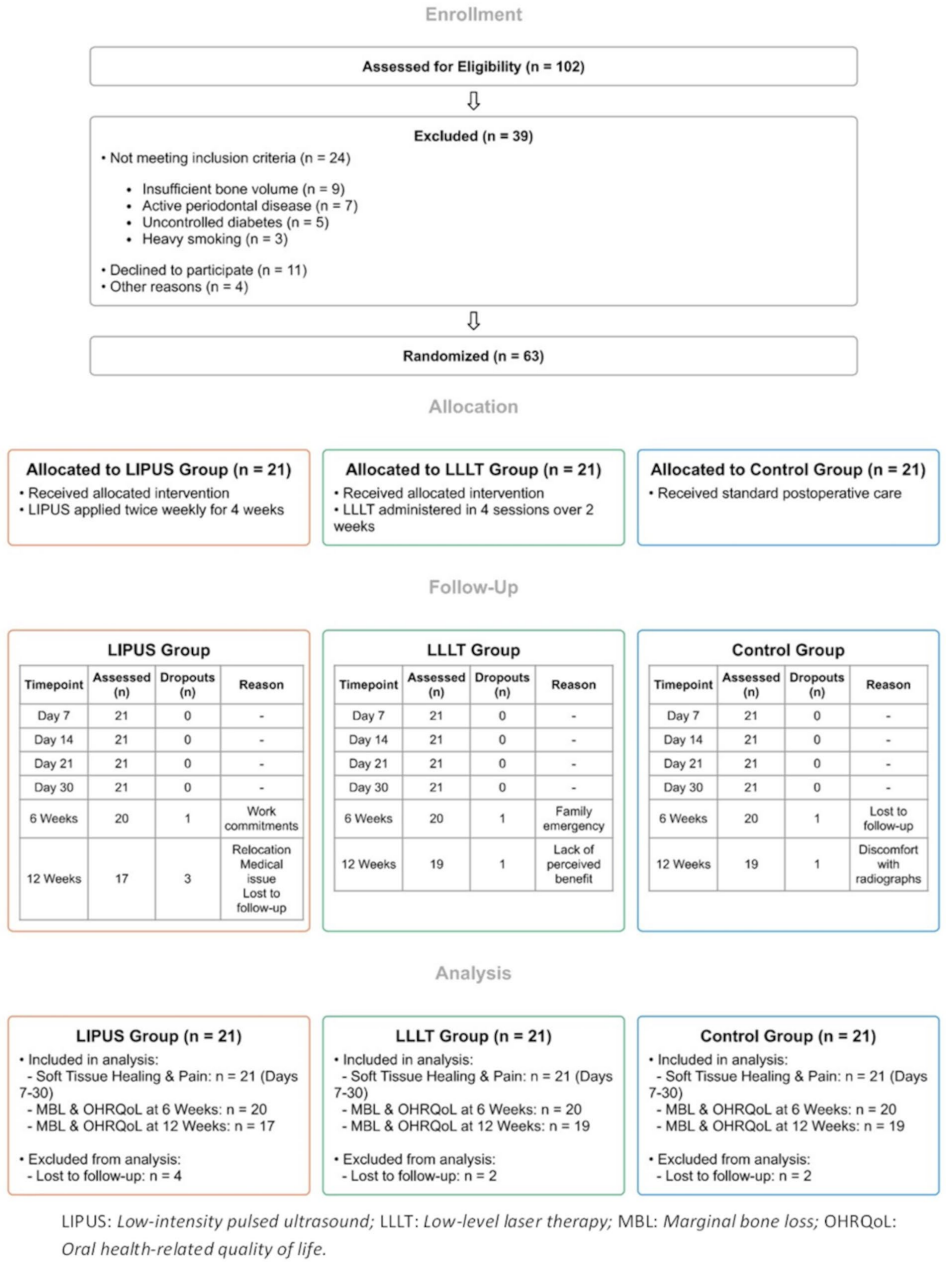
Flow of participants through the trial

Patient recruitment took place at the outpatient clinics of the Oral-Maxillofacial Surgery and Prosthodontics departments in multiple hospitals across Cairo via healthcare referrals and direct patient contact. Initial screening assessed eligibility per study criteria. Eligible patients received comprehensive study information with opportunities for clarification. The flow of participants through each stage of the trial is illustrated in Fig. 1.

The inclusion criteria were as follows: [1] Adults aged ≥ 18 years requiring dental implants in either the maxilla or mandible; [2] both male and female patients, to allow for gender comparisons; [3] non-smokers, as smoking has been shown to negatively impact implant success rates [32]; and [4] adequate bone volume and density at the implant site, defined as Class III or better according to the Lekholm and Zarb classification [33], as assessed clinically and radiologically.

Study exclusion criteria encompassed: [1] compromised medical status, particularly uncontrolled diabetes; [2] need for bone grafting at the intended implant location; [3] current facial radiotherapy or chemotherapy; [4] suboptimal oral hygiene; [5] previous TMJ disorders; and [6] concurrent oral or periodontal surgical procedures at implant sites.

### Sample size calculation

The number of study participants was determined by using G*Power 3.1.9.1; through power analysis based on the following input models; F tests– ANOVA; repeated measures, within-between interaction, given α, power, and effect size. Hence, we assumed 3 groups, 95% confidence interval, 0.05 α level, 80% power, and 0.331 effect size calculated from a previous study which resulted in 54 participants [34]. Considering possible losses, a 15% contingency was added to the number obtained, totaling 63 participants or 21 per group.

### Randomization & blinding

A randomized, single-blind study design was used to ensure unbiased treatment allocation. Eligible patients were randomly assigned to one of three groups: [1] LIPUS [2], LLLT, or [3] control, using a computer-generated randomization sequence with a 1:1:1 allocation ratio. The randomization sequence was generated by an independent statistician using a permuted block randomization method with a block size of six.

Allocation concealment was maintained using sealed, opaque, sequentially numbered envelopes, opened only after participants completed baseline assessments and provided informed consent. Due to the nature of the interventions, blinding of patients and treating clinicians was not feasible. However, outcome assessors were blinded to the patients’ group allocations.

To maintain blinding, all study-related documents were labeled with unique participant identification numbers rather than group assignments. The study coordinator kept a separate, confidential log linking the identification numbers to the group assignments, which was not accessible to the outcome assessors.

### Interventions

All implant procedures followed an established surgical protocol to ensure procedural consistency [35, 36]. The surgical sequence began with the administration of local anesthetic (2% lidocaine containing 1:100,000 epinephrine). Access to the recipient site was achieved through a midcrestal incision, followed by the reflection of a full-thickness mucoperiosteal flap. Critical anatomical structures were carefully identified and protected throughout the procedure. Implant site preparation was guided by a customized surgical stent. During placement, a minimum clearance of 1.5 mm was maintained between the implant and neighboring dentition to ensure adequate preservation of adjacent hard and soft tissues. The procedure concluded with precise flap repositioning and suturing to obtain tension-free primary closure.

Patients from all three groups received the standard postoperative care. In addition to the standard care, the low-intensity pulsed ultrasound (LIPUS) group received 8 treatment sessions (two sessions per week, 20 min each), while the low-level laser therapy (LLLT) group received 4 treatment sessions (on day 0, 3, 7, and 14 after surgery, 10 s per site). The control group received the standard postoperative care only. Interventions are described according to the template for intervention description and replication (TIDieR) guide [37].

#### Procedures

##### Low-intensity pulsed ultrasound (LIPUS) therapy

The LIPUS intervention was delivered using an ENRAF NONIUS, SONOPLUS 492 device (ENRAF NONIUS, The Netherlands, model number: EN-SP492). Patients receiving LIPUS therapy were positioned in a dental chair with their head supported by a headrest. The treatment area was prepared by applying a thin layer of intra-oral gel to the buccal aspect of the implant site to ensure efficient energy transfer. The LIPUS treatment was administered via an intraoral delivery system, utilizing a 0.8 cm^2^ probe positioned against the buccal surface of the implant region. The probe was placed in direct contact with the buccal attached gingival tissue, separated only by a thin layer of intraoral transmission gel that served as a coupling medium. LIPUS therapy was initiated 48 h post-surgery and administered twice weekly for 4 consecutive weeks, totaling 8 treatment sessions. Each 20-minute session utilized ultrasonic waves at 1 MHz frequency, delivering a power output of 20 mW and maintaining an intensity of 30 mW/cm2 with a static transducer position [38]. Patients were instructed to remain still and relax during the treatment. After each session, the treatment area was cleaned, and the patient was inspected for any complications.

##### Low-level laser therapy (LLLT)

For the LLLT intervention, a Chattanooga device, model 27,841 (Chattanooga, USA), equipped with a gallium-arsenide (GaAs) diode laser was used. The laser device emitted light at a wavelength of 850 nm and operated at a power of 200 mW in continuous wave mode [39].

Patients were seated in a dental chair with their head supported by a headrest. The mucous membrane surrounding the implant site was cleaned and dried before the application of the laser. The laser treatment was administered by applying the probe at 10 mm standoff distance from the soft tissue surrounding the implant, with the beam directed at six distinct points circumferentially around the implant site: mesiobuccally, distobuccal, midbuccal, midlingual, mesiolingual, and distolingual [40]. Each site was irradiated for 10 s, delivering a total energy of 6 J per session. Patients and therapists wore protective goggles during the treatment.

Both devices were calibrated before the start of the study and at regular intervals throughout the trial to ensure consistent output. Calibration was performed using the manufacturers’ recommended protocols and certified equipment.

##### Standard postoperative care (control group)

Patients in the control group received standard postoperative care [19], which included several components:

1. Extra-oral ice pack application: Patients were instructed to apply ice packs on the external surface of the face, over the implant area, for 20 min every 2 h during the first 48 h after surgery.
2. Oral hygiene maintenance: Patients were advised to maintain their daily oral hygiene routine, including gentle brushing and rinsing with a saltwater solution (1/2 teaspoon of salt in 8 ounces of warm water) after each meal and before bedtime.
3. Soft diet: Patients were recommended to consume a soft diet for one week after surgery to minimize mechanical stress on the implant site.
4. Antibiotics: As a prophylactic measure, patients were prescribed oral amoxicillin (500 mg), to be taken every 12 h for 7 days.
5. Pain and inflammation management: Patients received ibuprofen (400 mg) to manage pain and inflammation, with instructions to take one tablet every 8 h for 4 days.
6. Chlorhexidine rinse: Patients were instructed to rinse their mouth with a 0.12% chlorhexidine gluconate solution twice daily for 2 weeks to enhance plaque control and maintain oral hygiene.

#### Monitoring of adverse events and patient comfort

A standardized protocol monitored intervention-related adverse events. Patients reported discomfort, pain, or unusual symptoms directly to therapists or coordinators. Documentation included event severity, duration, and interventions taken. Therapists monitored patient feedback, adjusting treatment session or providing rest periods as needed to maintain comfort.

#### Compliance and adherence monitoring

Treatment compliance was monitored through comprehensive treatment logs documenting session details and protocol deviations. Patients maintained standardized diaries recording oral hygiene, diet, medication use, and adverse events. Adherence was promoted through appointment reminders and weekly follow-up calls. Each session included standardized questionnaires and oral examinations assessing compliance, hygiene practices, and complications.

Adherence criteria were: LIPUS - attendance of ≥ 75% sessions (6/8) with 20-minute duration; LLLT - completion of all 4 sessions with prescribed parameters; and home care - completion of ≥ 80% prescribed oral hygiene, dietary, and medication protocols (verified through patient diaries). Non-adherence was defined as failure to meet these criteria and analyzed as a binary outcome.

### Outcomes

#### Peri-implant marginal bone preservation

Peri-implant marginal bone preservation between the bone and implant surface was evaluated using high-resolution digital intraoral radiographs taken with an I-Sensor H2 Digital Intraoral X-ray Imaging System (Guilin Woodpecker Medical Instrument Co., Ltd., Guilin, China) equipped with a rated input of DC 5 V, 500 mA. Radiographs were taken using a paralleling technique with a Rinn film holder and a rigid film-object X-ray source, with settings of 60 kV and 7 mA.

To ensure standardized radiographic imaging across all follow-up visits, a customized positioning device was fabricated for each patient, incorporating a silicone registration material anchored to the existing teeth, with an integrated radiograph holder to maintain consistent beam angulation. The silicone index material was used to create a customized bite block, which helped to maintain the same position of the film and the X-ray tube head for each patient across different time points.

Marginal bone measurements were conducted using specialized imaging software (Vistasoft 2.4.13, Durr Dental Italy S.r.l) [41]. Two key parameters were evaluated:

- Radiographic implant length (IL): the linear measurement in millimeters from the coronal implant margin to the apical tip, measured along the implant’s central axis [42].
- Residual bone height at the mesial (MI) and distal (DI) aspects of the implant: the vertical distance in millimeters from the coronal implant margin to the first bone-to-implant contact [42].

For each implant, measurements were obtained in triplicate on both mesial and distal aspects, with mean values recorded for each side. A correction factor, calculated from the relationship between actual and radiographic implant dimensions, was applied to compensate for any image distortion [43]. The extent of marginal bone resorption (MBL) was determined by calculating the difference between initial post-surgical bone levels and those observed at the three-month follow-up [44]. Assessment of peri-implant marginal bone preservation was performed at three time points: immediately post-implantation, at the six-week interval, and at three months post-surgery.

#### Soft tissue healing

Soft tissue healing at the implant site was assessed using the Landry Healing Index (HI) immediately post-implant and on days 7, 14, 21, and 30 [45]. This assessment tool examines five key parameters: tissue coloration, granulation tissue presence, bleeding response to palpation, exudate formation, and epithelial integrity. The index utilizes a 1–5 scoring system, where the lowest score [1] represents compromised healing characterized by extensive gingival redness (exceeding 50%), palpation-induced bleeding, evident granulation tissue, failed epithelialization at incision margins with peripheral tissue loss, and presence of suppuration. Conversely, the highest score [5] denotes optimal healing outcomes, indicated by uniformly pink gingival tissue, absence of bleeding upon palpation, no granulation tissue formation, and completely epithelialized incision margins without exposed connective tissue. Superior healing is reflected by higher scores. The scores were recorded by a single calibrated examiner to ensure consistency.

#### Pain intensity

Implant site pain intensity was measured at 12 h post-implant and on days 7, 14, 21, and 30 using the Visual Analogue Scale (VAS), a validated, subjective measure for acute and chronic pain [45]. Patients were instructed to mark their pain intensity on a 10-cm ruler representing a continuum between “no pain” (0) and “worst pain” [10] at the time of assessment [46]. The VAS scores were collected by the patients themselves, and the examiner provided guidance on how to mark the scale correctly.

#### Oral Health-Related quality of life (OHRQoL)

OHRQoL was assessed using the Oral Health Impact Profile-14 (OHIP-14), a shortened version of the OHIP-49 [47]. The OHIP-14 is a widely used instrument to evaluate patients’ satisfaction and OHRQoL after receiving dental implant therapy. The questionnaire was administered in-person by the examiner immediately post-implant, at 6 weeks, and at 3 months [48]. The oral health-related quality of life assessment utilized the OHIP-14 questionnaire, which evaluates seven distinct dimensions: functional restrictions, discomfort/pain, psychological impact, physical limitations, mental well-being, social interactions, and overall life impediment. Each dimension comprises two questions, resulting in a comprehensive 14-item assessment tool. Participants responded using a five-point scale ranging from 0 (never) to 4 (very often). Individual domain scores were obtained by combining the relevant item responses, while the comprehensive OHIP-14 score was calculated as the sum of all domain scores, yielding a potential range of 0–56. An inverse relationship exists between the scores and quality of life, with elevated scores indicating diminished oral health-related quality of life [49].

### Follow-up protocol

A single calibrated, blinded examiner conducted all assessments following standardized protocols. Baseline measurements (day 0) included marginal bone levels (digital periapical radiographs), soft tissue condition (Landry Healing Index), and OHRQoL (OHIP-14 questionnaire), with pain first assessed via VAS at 12 h post-surgery. Early follow-ups (days 7, 14, 21, 30) evaluated soft tissue healing and pain levels, with all participants (*n* = 21 per group) completing these assessments without dropouts (Fig. 1).

Comprehensive evaluations at 6 and 12 weeks included marginal bone level radiographs, and OHRQoL assessments. At 6 weeks, one participant from each group missed follow-up (LIPUS: work commitments; LLLT: family emergency; control: lost to follow-up), leaving 20 participants per group. By 12 weeks, additional dropouts occurred in the LIPUS (4 total: relocation, medical issues, loss to follow-up), LLLT (2: perceived lack of benefit, loss to follow-up), and control groups (2: radiograph discomfort, loss to follow-up).

### Data analysis

ANOVA and chi-squared tests were used for baseline comparisons. Data distribution normality was assessed using Shapiro-Wilk test, and homogeneity by Levene’s test. For normally distributed data, MANOVA with repeated measures analyzed time intervals within groups, while MANOVA compared between groups. For non-normal distribution, Friedman and Wilcoxon Signed Ranks tests analyzed within-group differences, while Kruskal-Wallis and Mann-Whitney U tests compared between groups. Significance level was set at *p* < 0.05 (SPSS v25, IBM).

## Results

### Subject characteristics & adherence rate

Table 1 demonstrates comparable baseline characteristics across all groups. Demographics (mean age: 47.95–49.5 years; female predominance: 55–65%), clinical parameters (primary etiology: caries 40–45%), and implant specifications (maxilla: 45–60%; mandible: 40–55%; length: 12.14–13.16 mm; diameter: 3.66–3.82 mm) showed no significant differences (*p* > 0.05). Treatment adherence was high in all groups (LIPUS: 76%, LLLT: 86%, control: 90%; χ² = 1.66, *p* = 0.44; Supplementary File 2).

**Table 1:** Demographic, clinical, and implant characteristics of study participants

Characteristics	Group A (*n* = 21)	Group B (*n* = 21)	Group C (*n* = 21)	*p*-value†
Age (years)*	49.5 ± 8.06	47.95 ± 6.56	48.85 ± 5.86	**0.77‡**
Sex§				
Female	12 (57.1%)	11 (52.4%)	13 (61.9%)	**0.82**
Male	9 (42.9%)	10 (47.6%)	8 (38.1%)	
Duration of Edentulism (months)*	5.4 ± 1.8	6.3 ± 1.7	5.8 ± 1.6	**0.22‡**
Reason for Tooth Loss§				
Caries	10 (47.6%)	8 (38.1%)	9 (42.9%)	**0.17**
Periodontal Disease	5 (23.8%)	8 (38.1%)	9 (42.9%)	
Trauma	3 (14.3%)	5 (23.8%)	0 (0.0%)	
Failed Endodontic Treatment	3 (14.3%)	0 (0.0%)	3 (14.3%)	
Implant Location§				
Maxilla	13 (61.9%)	9 (42.9%)	11 (52.4%)	**0.47**
Mandible	8 (38.1%)	12 (57.1%)	10 (47.6%)	
Implant Dimensions*				
Length (mm)	13.16 ± 1.61	12.97 ± 1.91	12.14 ± 1.48	**0.13‡**
Diameter (mm)	3.82 ± 0.34	3.74 ± 0.18	3.66 ± 0.49	**0.07‡**

*Values are presented as mean ± standard deviation; §Values are presented as number (percentage); †Statistical significance set at *p* < 0.05; ‡One-way ANOVA; Chi-square test used for categorical variables

### Effect of treatment on MBL, OHIP-14, VAS and landry index

#### Within group comparison

Statistical analysis revealed significant decreases in mesial and distal MBL for groups A and B across all intervals (*p* < 0.001), with no significant changes in group C (*p* > 0.05). OHIP-14 scores decreased significantly in all groups at 6 weeks and 3 months versus day 0, and at 3 months versus 6 weeks (*p* < 0.001). VAS scores showed significant sequential decreases (12 h through 30 days) in all groups (*p* < 0.001), with groups A and C continuing to decrease between 21 and 30 days (*p* < 0.05), while group B remained stable (*p* > 0.05). Landry index demonstrated significant increases across sequential time points (0–30 days) in all groups (*p* < 0.05), with groups A and B showing increases between 14 and 21 days (*p* < 0.05), and groups A and C exhibiting increases between 21 and 30 days (*p* < 0.01) (Tables 2 and 3).

**Table 2:** Mean mesial MBL, distal MBL and OHIP at day 0, 6 weeks and 3 months of postoperative of group A, B and C

	Day 0	6 weeks	3 months	F-value	*p*-value
	mean ± SD	mean ± SD	mean ± SD		
**Mesial MBL(mm)**					
Group A	2.95 ± 1.59	0.94 ± 0.51^a^	0.44 ± 0.35 ^a, b^	39.45	0.001
Group B	2.79 ± 1.25	1.80 ± 0.68 ^a^	1.12 ± 0.41 ^a, b^	40.49	0.001
Group C	2.58 ± 1.16	2.35 ± 0.74 ^a^	2.23 ± 0.69	2.15	0.15
**F-value**	**0.39**	**23.75**	**63.55**		
	*p* = 0.68	*p* = 0.001	*p* = 0.001		
**Distal MBL (mm)**					
Group A	2.52 ± 0.97	0.93 ± 0.44 ^a^	0.44 ± 0.25 ^a, b^	93.81	0.001
Group B	2.31 ± 0.92	1.73 ± 0.63 ^a^	1.07 ± 0.27 ^a, b^	24.51	0.001
Group C	2.39 ± 0.80	2.24 ± 0.68	2.16 ± 0.52	2.52	0.11
**F-value**	0.30	25.56	110.13		
	*p* = 0.74	*p* = 0.001	*p* = 0.001		
**OHIP**					
Group A	43.20 ± 1.51	11.55 ± 1.23 ^a^	2.50 ± 1.24 ^a, b^	4040.53	0.001
Group B	42.90 ± 1.41	11.75 ± 1.29 ^a^	2.75 ± 1.33 ^a, b^	3936.49	0.001
Group C	42.15 ± 1.89	22.20 ± 1.36	9.80 ± 1.36	1944.76	0.001
**F-value**	2.23	441.15	199.90		
	*p* = 0.12	*p* = 0.001	*p* = 0.001		

SD, Standard deviation; MD, Mean difference; *p*-value, Probability value; ^a^ significant difference with day 0; ^b^ significant difference with 6 weeks

**Table 3:** Comparison of mesial MBL, distal MBL and OHIP at 6 weeks and 3 months postoperative between group A, B and C

	Group A vs. B					Group A vs. C					Group B vs. C				
	MD	95% CI		*p* value	ES	MD	95% CI		*p* value	ES	MD	95% CI		*p* value	ES
		LB	UB				LB	UB				LB	UB		
**6 weeks**															
Mesial MBL	-0.86	-1.36	-0.36	0.001	1.43	-1.41	-1.91	-0.91	0.001	2.22	-0.55	-1.05	-0.05	0.02	0.77
Distal MBL	-0.8	-1.26	-0.35	0.001	1.47	-1.31	-1.77	-0.87	0.001	2.29	-0.51	-0.97	-0.06	0.02	0.78
OHIP	-0.2	-1.19	0.79	0.87	0.16	-10.65	-11.64	-9.66	0.001	8.21	-10.45	-11.44	-9.46	0.001	7.88
**3 months**															
Mesial MBL	-0.68	-1.07	-0.30	0.001	1.78	-1.79	-2.18	-1.41	0.001	3.27	-1.11	-1.50	-0.72	0.001	1.96
Distal MBL	-0.63	-0.91	-0.35	0.001	2.42	-1.72	-2.00	-1.44	0.001	4.15	-1.09	-1.37	-0.81	0.001	4.15
OHIP	-0.25	-1.25	0.75	0.82	0.19	-7.3	-8.30	-6.30	0.001	5.61	-7.05	-8.05	-6.05	0.001	5.24

MD, Mean difference; CI, Confidence interval; LB, Lower bound; UB, Upper bound; *p* value, Probability value; EF, effect size

#### Between group comparison

Intergroup analysis (Table 4) revealed significantly lower MBL in group A versus groups B and C (*p* < 0.001), and in group B versus group C (*p* < 0.05) at both timepoints. OHIP-14 scores were significantly lower in groups A and B versus group C (*p* < 0.001), with no difference between A and B (*p* > 0.05). VAS scores (Table 5) showed no difference between groups A and B at 7, 21, and 30 days (*p* > 0.05), but both were significantly lower than group C (*p* < 0.001). At day 14, group B showed lowest VAS scores (*p* < 0.001). Landry index was highest in group B across all timepoints (*p* = 0.001), while groups A and C were comparable until day 30, when group A showed higher values (*p* < 0.05).

**Table 4:** Median values of VAS and Landay index at day 0, 12 h, 7, 14, 21 and 30 days postoperative of group A, B and C

	12 h	Day 7	Day 14	Day 21	Day 30	χ^2^ value	*p*-value
	Median (IQR)	Median (IQR)	Median (IQR)	Median (IQR)	Median (IQR)		
**VAS**							
Group A	10 (10, 9.25)	4 (5,4) ^a^	2 (3.75, 2) ^a, b^	0 (0.75, 0) ^a, b, c^	0 (0, 0) ^a, b, c, d^	79.02	0.001
Group B	10 (10, 9)	4 (4.75,4) ^a^	0 (2.0) ^a, b^	0 (0, 0) ^a, b, c^	0 (0, 0) ^a, b, c,^	76.80	0.001
Group C	10 (10, 10)	7 (7,6.25) ^a^	5 (5, 5) ^a, b^	3 (3.75, 3) ^a, b, c^	2 (2, 2) ^a, b, c, d^	78.45	0.001
**Kruskal-Wallis H**	**0.52**	**33.32**	**46.10**	40.33	46.65		
	*p* = 0.77	*p* = 0.001	*p* = 0.001	*p* = 0.001	*p* = 0.001		
	**Day 0**	**Day 7**	**Day 14**	**Day 21**	**Day 30**		
**Landay index**							
Group A	2 (2, 2)	2 (3,2) ^a^	3 (4, 3) ^a, b^	4 (4.75, 3) ^a, b, c^	5 (5, 4) ^a, b, c, d^	66.14	0.001
Group B	2 (2, 2)	4 (4, 3.25) ^a^	5 (5, 4) ^a, b^	5 (5, 5) ^a, b, c^	5 (5, 5) ^a, b, c,^	75.24	0.001
Group C	2 (2, 2)	2 (3,2) ^a^	3 (3, 3) ^a, b^	3 (4,3) ^a, b,^	4 (4, 4) ^a, b, c, d^	64.30	0.001
**Kruskal-Wallis H**	0	30.25	27.37	30.79	26.79		
	*p* = 1	*p* = 0.001	*p* = 0.001	*p* = 0.001	*p* = 0.001		

IQR, interquartile range; χ^2^: Chi squared value; *p*-value, Level of significance; ^a^ significant difference with 12 h/day 0; ^b^ significant difference with day 7. ^c^ significant difference with day 14; ^d^ significant difference with day 21

**Table 5:** Comparison of VAS and Landay index at 7, 14, 21 and 30 days postoperative between group A, B and C

	Group A vs. B		Group A vs. C		Group B vs. C	
	U-value	*p* value	U-value	*p* value	U-value	*p* value
**Day 7**						
VAS	189	0.70	32	0.001	29.5	0.001
Landay index	35	0.001	179	0.50	62.5	0.001
**Day 14**						
VAS	39	0.001	29.5	0.001	6	0.001
Landay index	44.5	0.001	171.5	0.36	44	0.001
**Day 21**						
VAS	159	0.07	26	0.001	21	0.001
Landay index	50	0.001	141.5	0.09	30	0.001
**Day 30**						
VAS	200	1	30	0.001	30	0.001
Landay index	110	0.001	132.5	0.03	30	0.001

U-value, Mann-Whitney test value, Probability value

**Fig. 2:**
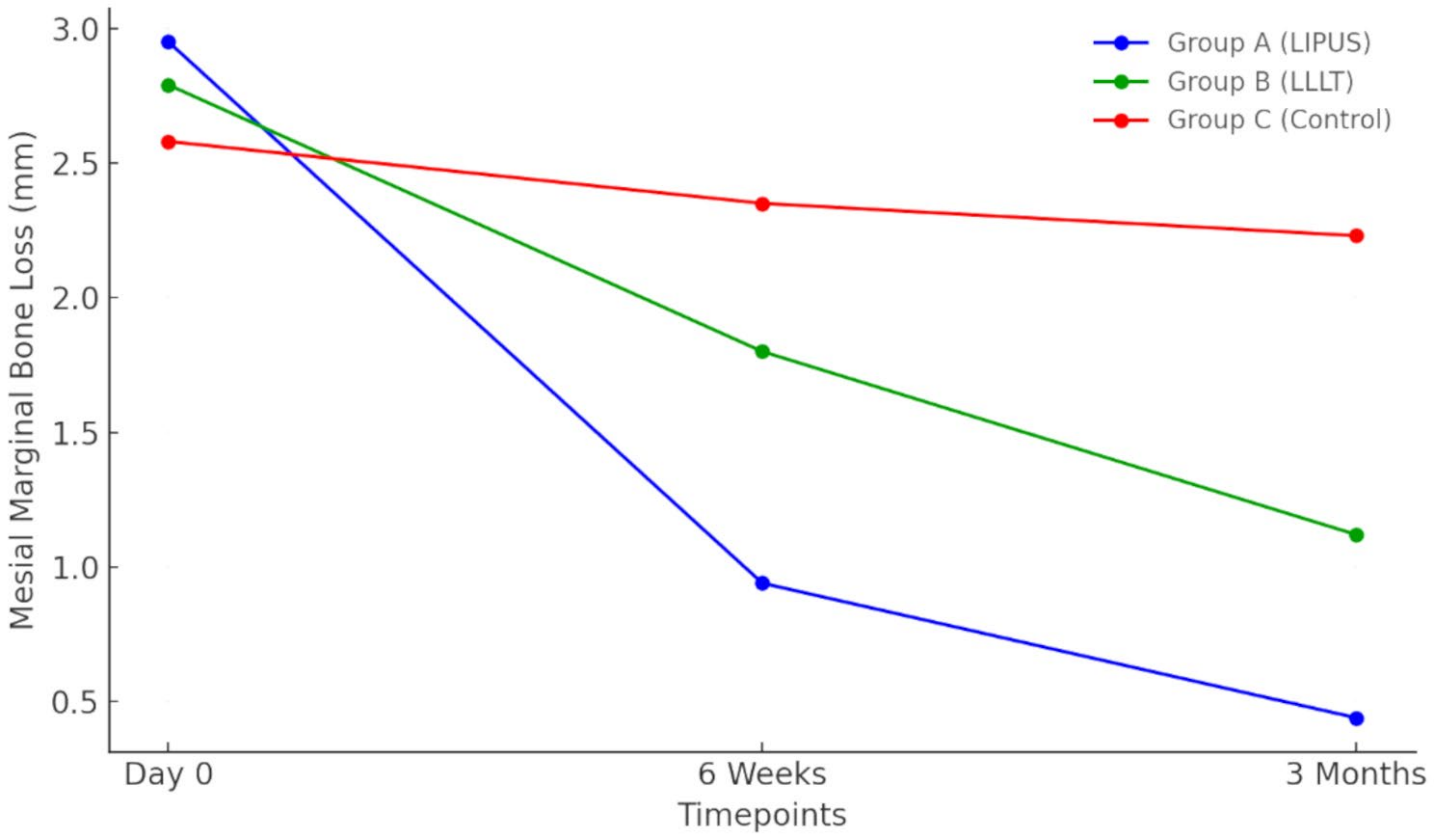
Mesial Marginal Bone Loss over Time from baseline to 3 months postoperatively

**Fig. 3:**
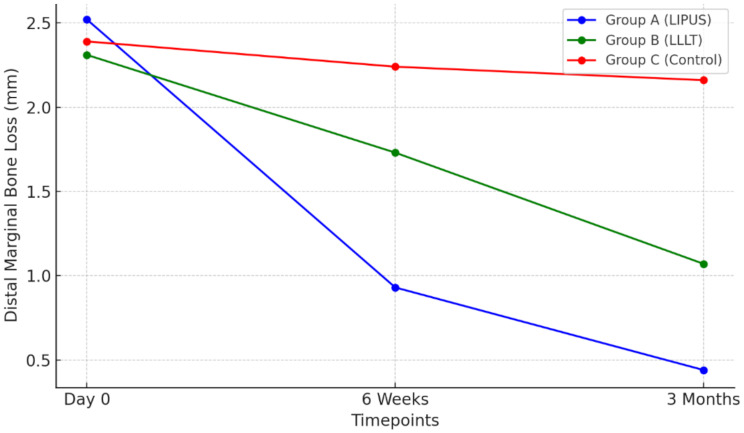
Distal Marginal Bone Loss over Time from baseline to 3 months postoperatively

**Fig. 4:**
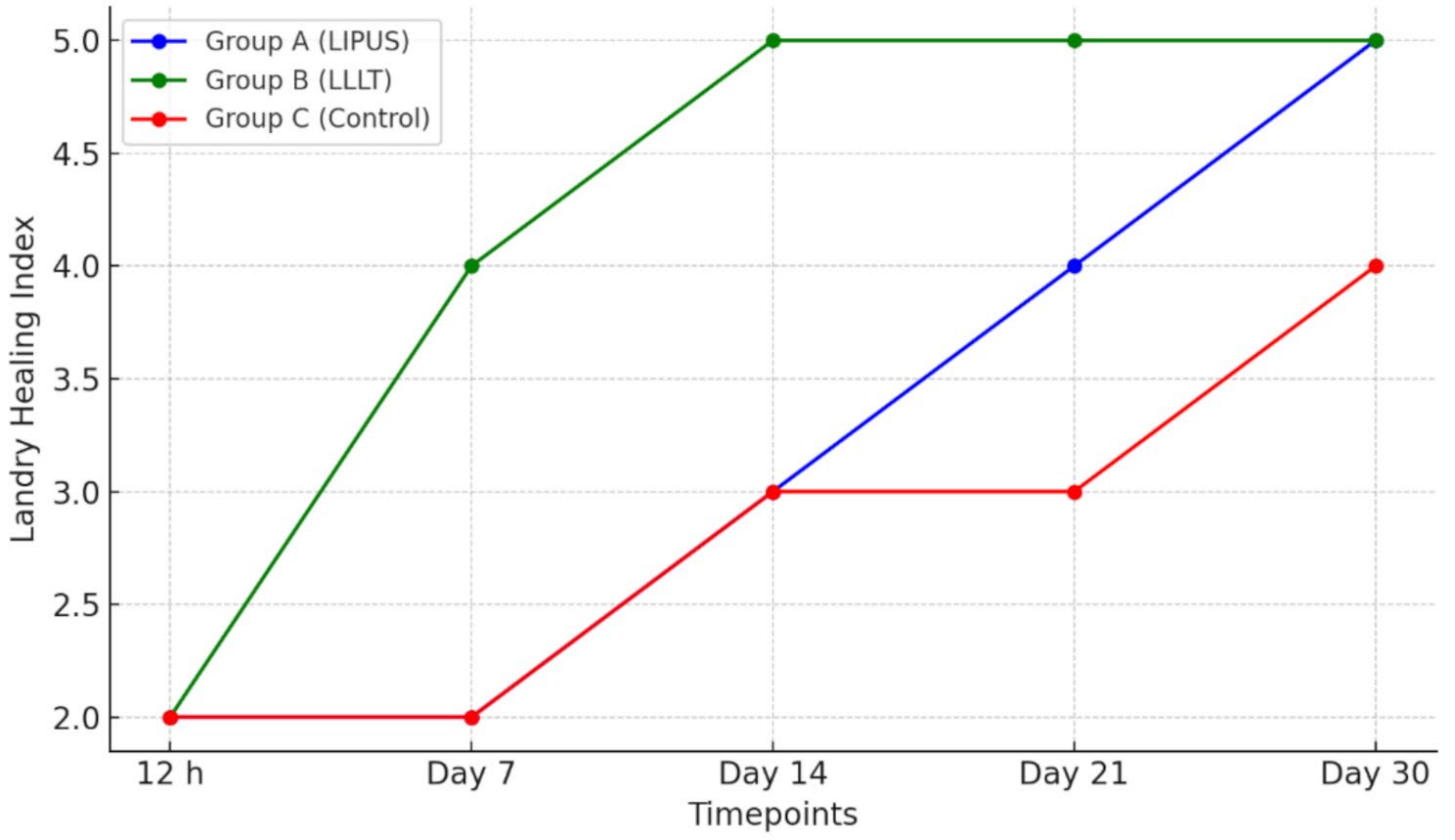
Landry Healing Index Scores over Time

**Fig. 5:**
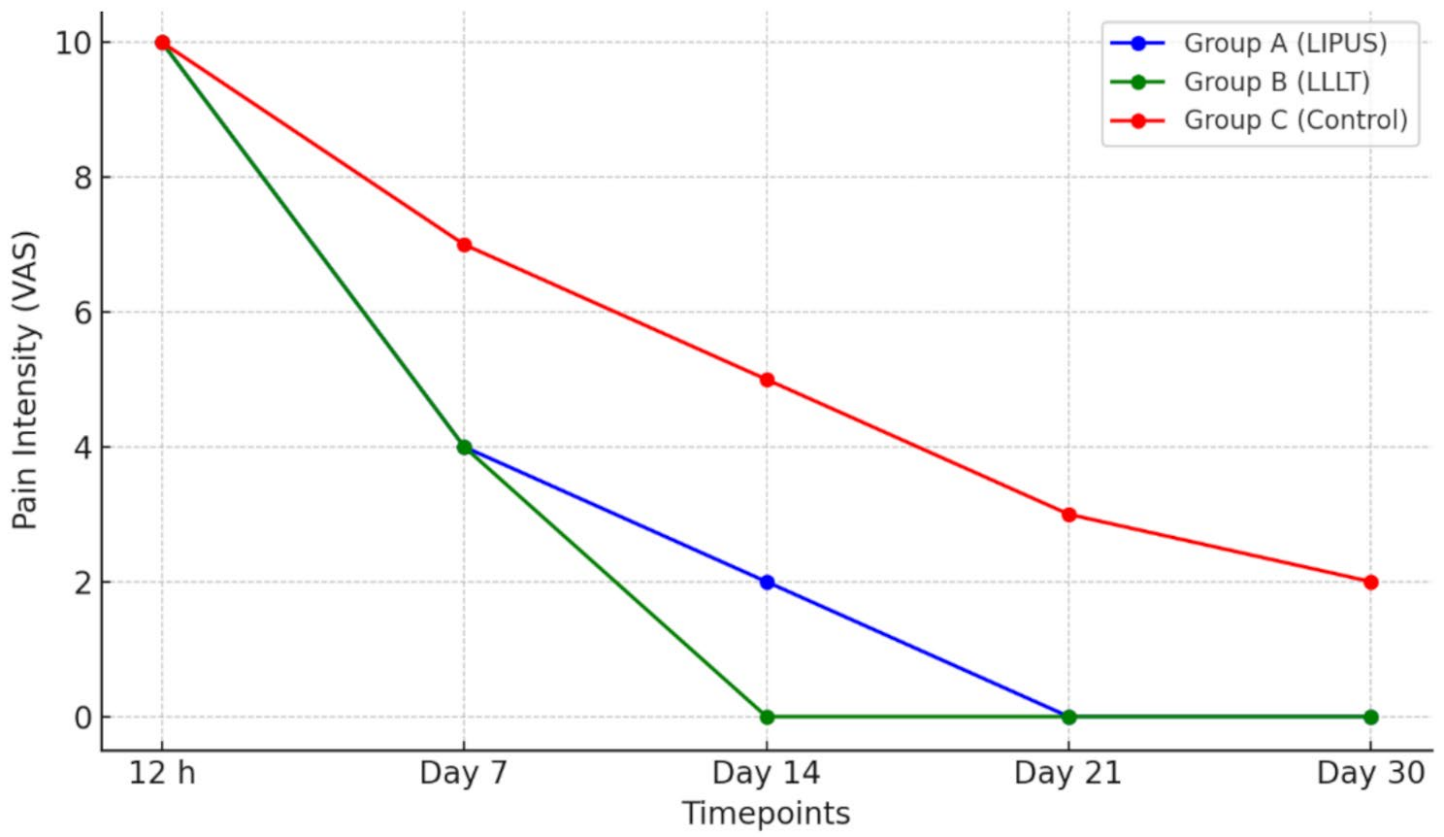
Pain Intensity Scores (VAS) over Time

**Fig. 6:**
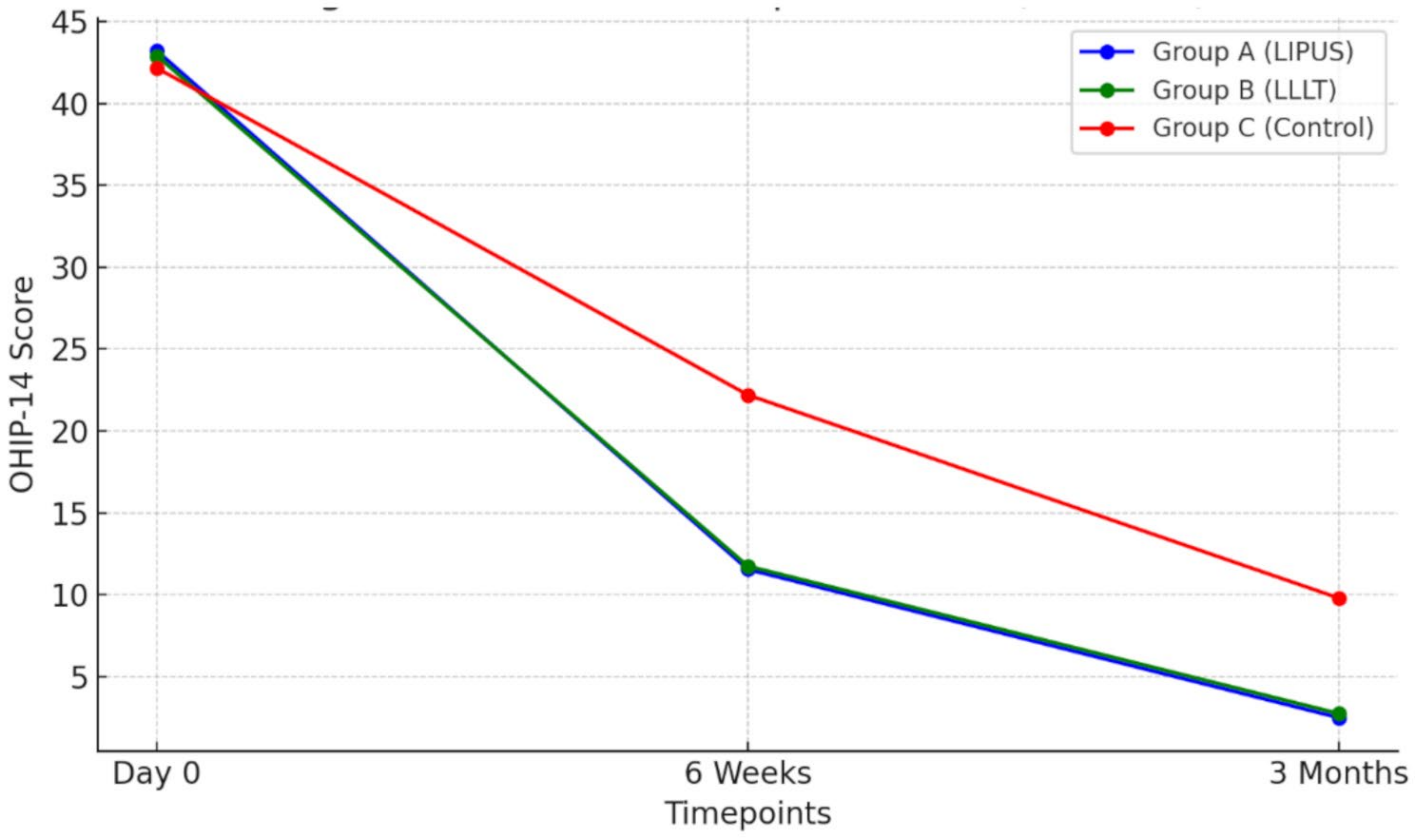
Oral Health Impact Profile (OHIP-14) over Time

Detailed analyses of outcomes at each specific time-point are provided in Figs. 2, 3, 4, 5 and 6 and Supplementary File 3.

## Discussion

This single-blind RCT compared LIPUS and LLLT effects on peri-implant marginal bone preservation, healing, pain, and OHRQoL. LIPUS showed superior marginal bone preservation at 6 weeks and 3 months, while LLLT achieved better soft tissue healing across all timepoints (7–30 days). Both therapies reduced pain and improved OHRQoL versus controls, demonstrating their potential to enhance implant surgery outcomes.

Our study revealed a significant reduction in marginal bone loss (MBL) in the LIPUS group compared to LLLT and standard care groups, with 0.44 mm MBL at 3 months for LIPUS versus 1.12 mm and 2.23 mm for LLLT and standard care, respectively. This suggests LIPUS effectively enhances peri-implant marginal bone preservation, supporting its potential as an adjunctive therapy in implant dentistry.

Our findings demonstrate both statistical significance and clinically meaningful improvements. LIPUS showed significantly reduced marginal bone loss (approximately 0.44 mm) compared to LLLT (1.12 mm) and control (2.23 mm). Clinically, the relevance of this reduction is substantial, as marginal bone loss greater than 1.5–2 mm within the first year post-implant placement is commonly associated with increased risk of implant complications or failure [50]. Early marginal bone loss exceeding 0.5 mm within the first six months is considered a critical predictor of ongoing bone loss and future peri-implant disease [51].

The observed pain relief in both LIPUS and LLLT groups exceeded the minimal clinically important difference (MCID) threshold typically cited in the literature (∼ 12 mm on a 100 mm VAS scale [52]). At 14 days postoperatively, LLLT patients reported almost complete pain relief (VAS = 0), while the control group still experienced moderate pain (VAS = 5), representing a 50-mm difference—far exceeding the minimal clinically relevant threshold. Similarly, OHIP-14 improvements surpassed the established minimal clinically important difference of 3–5 points [53].

Liu et al. [17] supports our findings in a rabbit model study (*n* = 24). They applied LIPUS (1.5 MHz, 30 mW/cm2, 20 min/day) to titanium implants in rabbit tibiae. LIPUS treatment significantly increased bone-implant contact by 36% and bone volume by 48%. These improvements correlate with our observed reduced marginal bone loss (MBL).

Translating these findings to humans, Abdulhameed et al. [18] corroborates our results in a clinical study (*n* = 22). They applied LIPUS (1.5 MHz, 30 mW/cm2, 20 min/session) before and after implant loading. Cone-beam CT (CBCT) analysis showed significantly increased marginal bone levels at six months, especially at the buccal site. CBCT provided precise marginal bone assessment, as noted by Bertossi et al. [54] in their review of cone-beam imaging in craniofacial medicine. This aligns with our reduced MBL findings of 68.14% at 6 weeks and 85.08% at 3 months post-implantation.

El-Fekey [55] studied LIPUS effects on implants in the esthetic zone (*n* = 10). LIPUS (1.5 MHz, 40 mW/cm2) was applied for 21 days. They found significantly increased implant stability and reduced crestal bone loss over one year. Their mean crestal bone loss of 0.6232 mm after one year is comparable to our reduced MBL results of 0.44 mm. However, the smaller sample size and different measurement time points should be considered when comparing results.

LIPUS enhances osseointegration primarily through mechanotransduction pathways that activate osteoblasts via integrin receptors and stretch-activated ion channels [56]. This mechanical stimulation increases cyclo-oxygenase-2 expression and prostaglandin E2 production, directly promoting osteoblast differentiation and mineral matrix deposition [57]. Additionally, LIPUS upregulates BMP-2, RUNX2, and Osterix expression, crucial transcription factors for osteogenesis [58].

The Landry Healing Index (LHI) results demonstrated that LLLT significantly improved soft tissue healing at 7-, 14-, 21-, and 30-days post-implant compared to LIPUS and control groups. At 30 days, the mean LHI scores were 4.6 ± 0.3, 3.9 ± 0.4, and 3.5 ± 0.5 for the LLLT, LIPUS, and control groups, respectively (*p* < 0.05).

These findings align with Palled et al. [30], who reported improved soft tissue healing parameters in LLLT-treated implant patients. While they used different assessment methods, their LLLT group showed significantly lower modified sulcus bleeding index scores and probing depths at 6 weeks and 3 months post-operatively, similar to our improved Landry index scores. Our LLLT protocol for 14 days postoperatively differs from Palled et al.‘s intermittent application. Despite these methodological differences, the consistent positive outcomes underscore LLLT’s efficacy in promoting soft tissue healing after dental implant surgery.

Pouremadi et al. [45] corroborated these findings in their study on LLLT effects after advanced implant surgeries. They observed significantly better wound healing scores on the 3rd, 7th, and 14th days post-surgery in the LLLT group. Our results mirror this trend, with Group B showing significantly higher Landry index scores from day 7 post-surgery.

Our study’s findings of significantly better outcomes with LLLT are supported by previous research. Camolesi et al. [59] conducted an RCT involving 30 patients who received immediate dental implants and were treated with LLLT (dual 630 nm and 808 nm, 4 J/cm² per point), reporting substantial improvements in soft tissue healing; nearly half of the LLLT group achieved excellent wound healing versus 18% in the control group.

Despite the general agreement, a few studies report more equivocal results, likely due to differences in laser parameters or assessment methods. For instance, one randomized trial by Collado-Murcia et al. [60] using a single intraoperative diode laser session (940 nm, 15 J/cm²) found no statistically significant improvement in early wound-healing index scores with LLLT at 1 week post-surgery. The comparable results might be due to insufficient laser dosage or assessment timing when natural healing was already progressing well. Studies using multiple LLLT applications (like ours) consistently show accelerated reduction in swelling, bleeding, and faster wound closure.

LLLT’s biostimulatory effects explain these improvements. It modifies inflammation, promotes angiogenesis, and stimulates fibroblast and keratinocyte activity, accelerating healing by improving tissue regeneration [45]. It increases ATP production and reactive oxygen species acting as secondary messengers that enhance cell migration and proliferation [26]. LLLT upregulates VEGF, TGF-β, and basic fibroblast growth factor, promoting angiogenesis and collagen synthesis in oral mucosa [61], while its anti-inflammatory effects through NF-κB pathway modulation primarily benefit soft tissue healing [62]. These mechanisms directly impact the tissue color and granulation tissue formation assessed in the Landry Healing Index.

VAS scores decreased significantly in all groups over time (*p* < 0.001). Both Group A (LIPUS) and Group B (LLLT) demonstrated superior pain control compared to Group C (control). By day 14, Group B reached a median VAS of 0, while Group A reached 2, both significantly lower than Group C at 5 (*p* < 0.001). On day 7, both LIPUS and LLLT groups showed equally effective pain reduction (median VAS 4) compared to the control (median VAS 7) (*p* < 0.001).

These findings align with Gopalan et al. [63], who reported significant pain reduction in LIPUS-treated patients after mandibular fracture fixation. Similarly, Dhole et al. [64] reported that LLLT significantly reduced post-operative pain and crestal bone loss around dental implants. They observed lower VAS pain scores in the LLLT group on days 2 and 4 and reduced mean crestal bone loss at 4 and 6 months compared to the control group. Our results suggest that both LIPUS and LLLT effectively reduce post-operative pain, with LLLT potentially offering slightly faster pain relief in the second week.

OHIP-14 scores decreased significantly in all groups over time (*p* < 0.001). At 3 months, both LIPUS and LLLT groups showed significantly lower OHIP-14 scores (2.50 ± 1.24 and 2.75 ± 1.33, respectively) compared to the control group (9.80 ± 1.36) (*p* < 0.001). Notably, there was no significant difference between LIPUS and LLLT groups (*p* = 0.82), suggesting similar efficacy in improving OHRQoL. Our OHIP-14 findings align with those of Souza et al. [65], who reported significant improvements in OHRQoL following LLLT application in oral surgery. While their study focused on molar extractions rather than implants, they similarly found that LLLT groups showed significantly lower OHIP-14 scores compared to controls at early follow-up periods. At 30 days, their LLLT group showed mean OHIP-14 scores of 0.70 ± 0.67, comparable to our findings of improved OHRQoL in the LLLT group at 3 months.

Study strengths include randomized controlled design, parallel-group allocation, objective/subjective outcome measures, and rigorous methodology, enhancing internal validity. However, this study has several limitations. Our single-center design restricts external validity to diverse clinical environments. The modest sample size (*n* = 63) limits statistical power for detecting smaller effect sizes and subgroup differences.

The three-month follow-up period, while sufficient for initial assessment, inadequately captures critical long-term outcomes in dental implant therapy. This brief timeframe prevents evaluation of implant survival rates and late complications like peri-implantitis, which typically manifest beyond our observation period. Standard implant survival rates range from 90 to 95% over 5–10 years [66], requiring longer monitoring to determine if early benefits persist.

Our single-blind methodology potentially introduces bias, particularly for subjective measures. While outcome assessors remained blinded, patients and clinicians knew their treatment allocation, possibly inflating effect sizes [67].

The study’s use of implants with similar design characteristics and surface topologies enhanced internal consistency but limited generalizability. Rego et al. [68] demonstrated that variations in implant design and surface properties significantly affect osseointegration and soft tissue healing outcomes, suggesting our results may not apply to all implant systems. Potential confounding factors, including variations in oral hygiene practices, dietary habits, and individual healing capacity, may have influenced outcomes despite randomization. While our study demonstrated significant effects of LIPUS on peri-implant marginal bone preservation, we acknowledge that direct assessment of osseointegration would require histological analysis, which was not feasible in this clinical study.

Importantly, we did not assess cost-effectiveness despite its critical relevance to clinical implementation. LIPUS devices ($1,300-$5,000) [69] and LLLT equipment represent substantial investments requiring economic justification, especially considering conventional implant therapy already achieves 90–95% success rates without adjunctive treatments [66]. The marginal improvement from these technologies must offset their costs to justify routine implementation. While orthopedic research suggests LIPUS can be cost-effective by preventing revision surgeries in fracture non-unions—saving approximately £2,400 per patient [70]—equivalent analyses in dental implantology are lacking.

Clinical implications support integrating both therapies into postoperative protocols, with LIPUS preferred for osseointegration and LLLT for soft tissue healing. Recommended protocol: LIPUS twice weekly for 4 weeks (8 sessions total, starting day 2 post-surgery); LLLT at days 0, 3, 7, and 14 post-implant using specified parameters. Implementation requires patient education regarding benefits and continued oral hygiene monitoring for optimal outcomes.

### Future directions

Future research priorities include: multi-center trials with larger cohorts and extended follow-up to assess long-term outcomes; optimization of treatment protocols; evaluation of patient-specific factors (oral hygiene, systemic conditions) on therapeutic efficacy; and cost-effectiveness analysis versus standard care.

## Conclusion

LIPUS and LLLT significantly enhanced peri-implant marginal bone level maintenance and soft tissue healing in dental implant patients compared to standard care. LIPUS was more effective for preserving marginal bone levels, while LLLT excelled in soft tissue healing. The study’s findings suggest that incorporating these interventions into postoperative protocols can optimize treatment outcomes and patient satisfaction.

## Electronic supplementary material

Below is the link to the electronic supplementary material.

Supplementary Material 1

## Data Availability

The datasets used and/or analysed during the current study are available from the corresponding author on reasonable request.

## Declarations

### Peer-review

The authors will comply with the editor’s decision on this matter.

## Abbreviations

LIPUS: Low-Intensity Pulsed Ultrasound
LLLT: Low-Level Laser Therapy
RCT: Randomized Controlled Trial
OHRQoL: Oral Health-Related Quality of Life
MBL: Marginal Bone Loss
OHIP-14: Oral Health Impact Profile-14
VAS: Visual Analogue Scale
HI: Healing Index
IL: Implant Length
MI: Mesial Implant
DI: Distal Implant
CONSORT: Consolidated Standards of Reporting Trials
TMJ: Temporomandibular Joint
CAD/CAM: Computer-Aided Design/Computer-Aided Manufacturing
TIDieR: Template for Intervention Description and Replication
GaAs: Gallium-Arsenide
ANOVA: Analysis of Variance
MANOVA: Multivariate Analysis of Variance
LHI: Landry Healing Index
