# Supplementary material for: Low intensity pulsed ultrasound versus low-level laser therapy on peri-implant marginal bone preservation and soft tissue healing following dental implant surgery: a randomized controlled trial
Source: Head Face Med. 2025 Apr 23;21:29. doi: 10.1186/s13005-025-00502-z (PMC12016225; doi:10.1186/s13005-025-00502-z)
Supplement: Supplementary file 1 — Supplementary Material 1 [file 13005_2025_502_MOESM1_ESM.doc]

**Supplementary file 1: CONSORT checklist.**

| Section/Topic | Item No | Checklist item | Reported on page No |
| --- | --- | --- | --- |
| Title and abstract | | | |
|  | 1a | Identification as a randomised trial in the title | 1 |
|  | 1b | Structured summary of trial design, methods, results, and conclusions (for specific guidance see CONSORT for abstracts) | 2-3 |
| Introduction | | | |
| Background and objectives | 2a | Scientific background and explanation of rationale | 3-4 |
|  | 2b | Specific objectives or hypotheses | 4 |
| Methods | | | |
| Trial design | 3a | Description of trial design (such as parallel, factorial) including allocation ratio | 3, 6 |
|  | 3b | Important changes to methods after trial commencement (such as eligibility criteria), with reasons | NA |
| Participants | 4a | Eligibility criteria for participants | 5 |
|  | 4b | Settings and locations where the data were collected | 4-5 |
| Interventions | 5 | The interventions for each group with sufficient details to allow replication, including how and when they were actually administered | 7-10 |
| Outcomes | 6a | Completely defined pre-specified primary and secondary outcome measures, including how and when they were assessed | 10-13 |
|  | 6b | Any changes to trial outcomes after the trial commenced, with reasons | NA |
| Sample size | 7a | How sample size was determined | 6 |
|  | 7b | When applicable, explanation of any interim analyses and stopping guidelines | NA |
| Randomisation: |  |  |  |
| Sequence generation | 8a | Method used to generate the random allocation sequence | 6 |
|  | 8b | Type of randomisation; details of any restriction (such as blocking and block size) | 6 |
| Allocation concealment mechanism | 9 | Mechanism used to implement the random allocation sequence (such as sequentially numbered containers), describing any steps taken to conceal the sequence until interventions were assigned | 6-7 |
| Implementation | 10 | Who generated the random allocation sequence, who enrolled participants, and who assigned participants to interventions | 4-7 |
| Blinding | 11a | If done, who was blinded after assignment to interventions (for example, participants, care providers, those assessing outcomes) and how | 6-7 |
|  | 11b | If relevant, description of the similarity of interventions | 7 |
| Statistical methods | 12a | Statistical methods used to compare groups for primary and secondary outcomes | 13 |
|  | 12b | Methods for additional analyses, such as subgroup analyses and adjusted analyses | NA |
| Results | | | |
| Participant flow (a diagram is strongly recommended) | 13a | For each group, the numbers of participants who were randomly assigned, received intended treatment, and were analysed for the primary outcome | 13, 24 |
|  | 13b | For each group, losses and exclusions after randomisation, together with reasons | 13, 24, supp file 2 |
| Recruitment | 14a | Dates defining the periods of recruitment and follow-up | 4 |
|  | 14b | Why the trial ended or was stopped | NA |
| Baseline data | 15 | A table showing baseline demographic and clinical characteristics for each group | 24 |
| Numbers analysed | 16 | For each group, number of participants (denominator) included in each analysis and whether the analysis was by original assigned groups | Supp file 2 |
| Outcomes and estimation | 17a | For each primary and secondary outcome, results for each group, and the estimated effect size and its precision (such as 95% confidence interval) | 13-14, 25-28 |
|  | 17b | For binary outcomes, presentation of both absolute and relative effect sizes is recommended | NA |
| Ancillary analyses | 18 | Results of any other analyses performed, including subgroup analyses and adjusted analyses, distinguishing pre-specified from exploratory | NA |
| Harms | 19 | All important harms or unintended effects in each group (for specific guidance see CONSORT for harms) | - |
| Discussion | | | |
| Limitations | 20 | Trial limitations, addressing sources of potential bias, imprecision, and, if relevant, multiplicity of analyses | 17 |
| Generalisability | 21 | Generalisability (external validity, applicability) of the trial findings | 17-18 |
| Interpretation | 22 | Interpretation consistent with results, balancing benefits and harms, and considering other relevant evidence | 14-17 |
| Other information | | |  |
| Registration | 23 | Registration number and name of trial registry | 5 |
| Protocol | 24 | Where the full trial protocol can be accessed, if available | - |
| Funding | 25 | Sources of funding and other support (such as supply of drugs), role of funders | - |

Citation: Schulz KF, Altman DG, Moher D, for the CONSORT Group. CONSORT 2010 Statement: updated guidelines for reporting parallel group randomised trials. BMC Medicine. 2010;8:18.
© 2010 Schulz et al. This is an Open Access article distributed under the terms of the Creative Commons Attribution License (<http://creativecommons.org/licenses/by/2.0>), which permits unrestricted use, distribution, and reproduction in any medium, provided the original work is properly cited.

*We strongly recommend reading this statement in conjunction with the CONSORT 2010 Explanation and Elaboration for important clarifications on all the items. If relevant, we also recommend reading CONSORT extensions for cluster randomised trials, non-inferiority and equivalence trials, non-pharmacological treatments, herbal interventions, and pragmatic trials. Additional extensions are forthcoming: for those and for up-to-date references relevant to this checklist, see [www.consort-statement.org](http://www.consort-statement.org/).

**Supplementary file 2: Treatment protocol adherence across study groups.**

| **Treatment Group** | **Adherence Status** | | **Total (n)** | **χ² test**  **(p-value)** |
| --- | --- | --- | --- | --- |
|  | **Adhered n (%)** | **Non-adherent n (%)** |  |  |
| **LIPUS** | 16 (76.2) | 5 (23.8) | 21 | 1.66 (**0.44**) |
| **LLLT** | 18 (85.7) | 3 (14.3) | 21 |  |
| **Control** | 19 (90.5) | 2 (9.5) | 21 |  |
| **Total** | **53 (84.1)** | **10 (15.9)** | **63** | **-** |

*Note: Values are presented as numbers and percentages. Statistical significance set at p < 0.05.*

Supplementary file 3: Detailed analyses of outcomes at each specific time-point

***Within-group comparisons:***

- **Marginal Bone Loss (MBL)**: Significant decreases in mesial and distal marginal bone loss were observed in both LIPUS and LLLT groups (p < 0.001). In Group A (LIPUS), mean mesial MBL significantly decreased from 2.95 ± 0.97 mm (day 0) to 0.93 ± 0.44 mm (6 weeks), and further reduced to 0.44 ± 0.25 mm at 3 months. Similarly, distal MBL decreased from 2.52 ± 0.97 mm to 0.93 ± 0.44 mm at 6 weeks, and further improved to 0.44 ± 0.25 mm at 3 months (p < 0.001). Group B (LLLT) also showed significant mesial MBL reduction from 2.79 ± 1.25 mm at baseline to 1.80 ± 0.68 mm at 6 weeks, and further to 1.12 ± 0.41 mm at 3 months (p < 0.001). Group C (control) showed no significant MBL changes over time (p > 0.05). Significant reductions in distal MBL were similarly noted in LIPUS and LLLT groups (p < 0.001), with no significant changes in the control group (Table 2).
- **Soft Tissue Healing (Landry Healing Index)**: Landry index scores indicated significant soft tissue healing improvements across all groups over the 30-day period (p < 0.001). The LLLT group consistently displayed superior healing scores at all follow-up time points (median Landry Index = 4 at day 7, reaching maximum scores of 5 by day 14 and maintained until day 30), significantly higher than both LIPUS and control groups (p < 0.001). Group A showed moderate improvements (median = 3 by day 14, improving further to 4 at day 21 and 5 at day 30), while the control group demonstrated comparatively slower progression, reaching a median Landry score of only 4 by day 30 (Tables 4 and 5).
- **Pain Intensity (VAS)**: All groups showed significant sequential decreases in pain intensity over the follow-up period (p < 0.001). Median VAS scores decreased notably from day 0 (VAS=10) to day 7 (Group A: 4, Group B: 4, Group C: 7), further reducing significantly at day 14, with Group B reaching near-zero levels of pain earlier (VAS median = 0), compared to Group A (median = 2) and Group C (median = 5). By day 21, pain in the control group remained significantly higher (median VAS = 3), while both LIPUS and LLLT groups achieved negligible levels (p < 0.001). At day 30, pain was effectively resolved in both intervention groups, remaining significantly lower than the control group (p < 0.001, Tables 4 and 5).
- **Oral Health-Related Quality of Life (OHIP-14)**: OHIP-14 scores improved significantly in all groups over time (p < 0.001). In Group A, OHIP-14 scores reduced from 43.20 ± 1.51 at baseline to 11.55 ± 1.51 at 6 weeks, further improving to 2.50 ± 1.24 at 3 months (p < 0.001). Similarly, Group B improved from 42.15 ± 1.89 to 22.20 ± 1.36 at 6 weeks, and 9.80 ± 1.36 at 3 months (p < 0.001). The control group demonstrated less pronounced but significant improvements (Table 2).

***Between-group comparisons***

- **Marginal Bone Loss*:*** Intergroup analysis confirmed significantly better peri-implant bone preservation with LIPUS compared to LLLT and control groups at both 6-week and 3-month evaluations (p < 0.001). At 3 months, Group A showed the lowest MBL (mean mesial MBL=0.44 ± 0.35 mm), significantly better than both Group B (1.12 ± 0.41 mm; p < 0.05) and Group C (2.23 ± 0.69 mm; p < 0.001).
- **Landry Healing Index:** The Landry index revealed that LLLT had significantly superior soft tissue healing across all follow-up intervals (days 7, 14, 21, and 30; p = 0.001). Differences between LIPUS and control groups became significant by day 30, favoring LIPUS over the control (p < 0.05), but both still lagged behind LLLT (Table 5).
- **Pain Intensity (VAS):** VAS scores showed no significant difference between LIPUS and LLLT groups at days 7, 21, and 30 (p > 0.05), though both groups significantly outperformed controls (p < 0.001). At day 14, LLLT showed significantly superior pain relief compared to both other groups (p < 0.001).
- **Oral Health-Related Quality of Life (OHIP-14)*:*** OHIP-14 scores at 6 weeks and 3 months were significantly lower (better) in both treatment groups compared to the control group (p < 0.001). No significant differences were observed between LIPUS and LLLT groups at either time point (Table 3).
